# Supplementary material for: Association between oral microbiome and seven types of cancers in East Asian population: a two-sample Mendelian randomization analysis
Source: Front Mol Biosci. 2023 Nov 21;10:1327893. doi: 10.3389/fmolb.2023.1327893 (PMC10702768; doi:10.3389/fmolb.2023.1327893)
Supplement: Supplementary file 1 [file DataSheet1.ZIP › Supplementary Materials/MR plots for tongue/tongue═╝/Lung cancer/pheno.3150_to_lung cancer_leave_one_out.pdf]

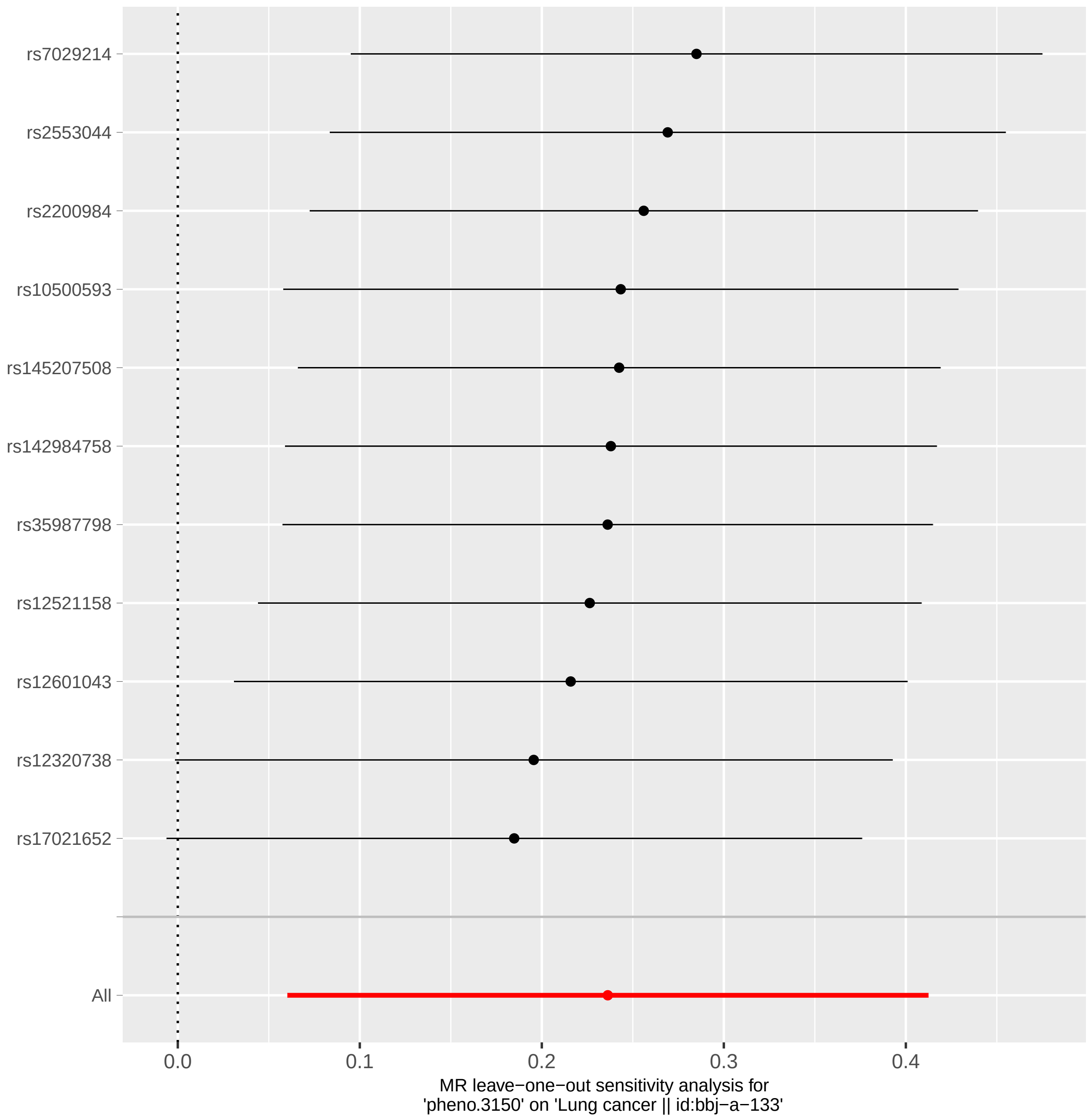
rs7029214
rs2553044
rs2200984
rs10500593
rs145207508
rs142984758
rs35987798
rs12521158
rs12601043
rs12320738
rs17021652
All
0.0
0.1
0.2
0.3
0.4
MR leave−one−out sensitivity analysis for
'pheno.3150' on 'Lung cancer || id:bbj−a−133'
